# The Pathological Society

**Published:** 1902-12-06

**Authors:** 


### THE PATHOLOGICAL SOCIETY.

At a recent meeting of the Pathological Society two communications were made which are of considerable interest as showing how with increasing intercommunication between this and other countries we must expect to meet more and more with diseases which have generally been regarded as foreign to our shores. The first of these was made by Dr. J. S. Haldane, who described an outbreak of anæmia in the Dolcoath mine in Cornwall, which had been traced to infection by the Ankylostoma Duodenale. This outbreak had been in progress for probably seven or eight years. The disease had evidently been spread by carelessness in the disposal of excreta. These were deposited anywhere in the mine and were carried by the boots of the miners on to the ladders and thus to their hands, and as the men took their dinners in the mine the circuit was thus completed. It did not seem that the disease was conveyed by water. The infection was very widespread, the ova being found in the fæces even of apparently healthy men. The disease appeared to have been introduced from abroad, many of the miners having worked in foreign mines. Mr. Boycott showed that, contrary to the common belief, moisture hindered the development of the ova. At a temperature of 54° Fahr. these remained unchanged, but at a temperature of 90° Fahr. they developed into worms in from two to four days. How extensively the disease had spread might be judged from the fact that out of 11 samples of fæces which he had examined 10 were swarming with embryos, and the remaining one, which had been more recently passed, contained a considerable number of ova.

The other communication referred to was one by Dr. Patrick Manson, C.M.G., in which he reported two cases of trypansoma occurring in the blood of man. Dr. Manson seemed to think that although there was no doubt about the presence of a protozoon the malady was not the ordinary "fly disease," since the blood injected into animals susceptible to the latter did not give rise to that disease.

